# Expression Profiles of Vpx/Vpr Proteins Are Co-related with the Primate Lentiviral Lineage

**DOI:** 10.3389/fmicb.2016.01211

**Published:** 2016-08-03

**Authors:** Yosuke Sakai, Ariko Miyake, Naoya Doi, Hikari Sasada, Yasuyuki Miyazaki, Akio Adachi, Masako Nomaguchi

**Affiliations:** ^1^Department of Microbiology, Tokushima University Graduate School of Medical ScienceTokushima, Japan; ^2^Laboratory of Molecular Immunology and Infectious Disease, Joint Faculty of Veterinary Medicine, Yamaguchi UniversityYamaguchi, Japan; ^3^Department of Microbiology and Cell Biology, Tokyo Metropolitan Institute of Medical ScienceTokyo, Japan

**Keywords:** HIV-2, SIV, Vpx, Vpr, PPM

## Abstract

Viruses of human immunodeficiency virus type 2 (HIV-2) and some simian immunodeficiency virus (SIV) lineages carry a unique accessory protein called Vpx. Vpx is essential or critical for viral replication in natural target cells such as macrophages and T lymphocytes. We have previously shown that a poly-proline motif (PPM) located at the C-terminal region of Vpx is required for its efficient expression in two strains of HIV-2 and SIVmac, and that the Vpx expression levels of the two clones are significantly different. Notably, the PPM sequence is conserved and confined to Vpx and Vpr proteins derived from certain lineages of HIV-2/SIVs. In this study, Vpx/Vpr proteins from diverse primate lentiviral lineages were experimentally and phylogenetically analyzed to obtain the general expression picture in cells. While both the level and PPM-dependency of Vpx/Vpr expression in transfected cells varied among viral strains, each viral group, based on Vpx/Vpr amino acid sequences, was found to exhibit a characteristic expression profile. Moreover, phylogenetic tree analyses on Gag and Vpx/Vpr proteins gave essentially the same results. Taken together, our study described here suggests that each primate lentiviral lineage may have developed a unique expression pattern of Vpx/Vpr proteins for adaptation to its hostile cellular and species environments in the process of viral evolution.

## Introduction

**FIGURE 1:**
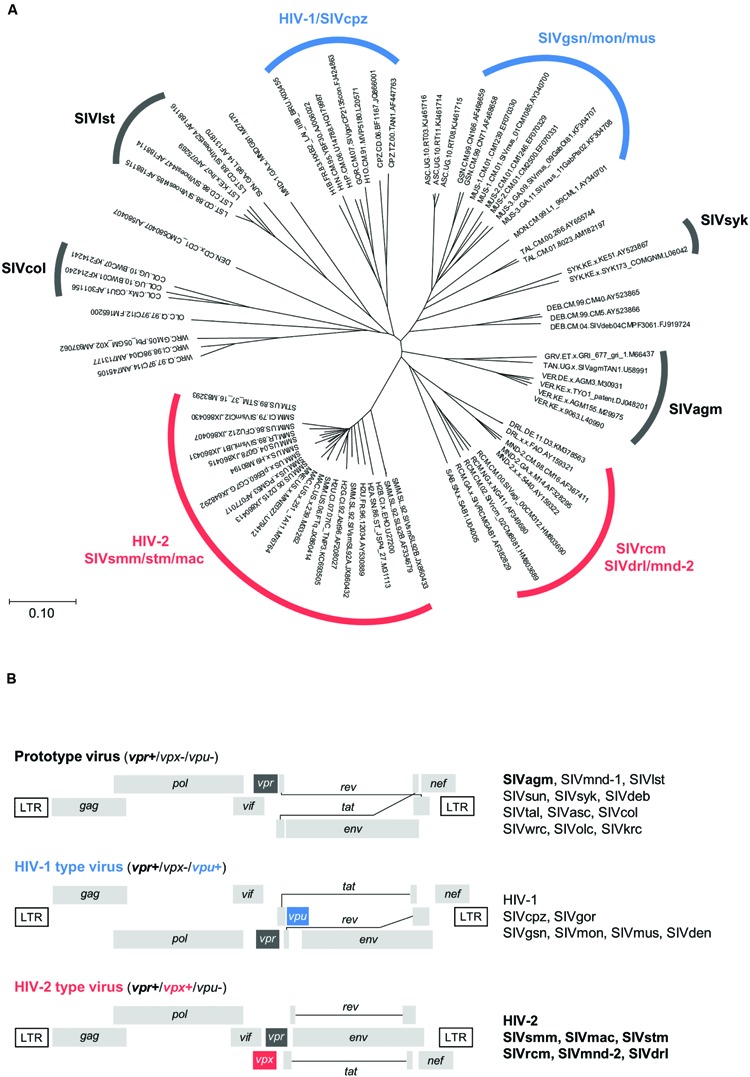
**Evolutionary relationship and genome structure of various HIV/SIVs. (A)** Phylogeny of HIV/SIVs. The unrooted phylogenetic tree shown was inferred by the neighbor-joining method using amino acid sequences of the entire Gag polyprotein. Amino acid sequences in the HIV Sequence Compendium (http://www.hiv.lanl.gov) were used to generate the tree. Scale bar represents the genetic distance. Eight major viral lineages (Peeters and Courgnaud, 2002; Gordon et al., 2005) are marked as shown. Virus clones not yet classified into the lineage groups remain unmarked. Three genome types (Fujita et al., 2010) are indicated by black (prototype), blue (HIV-1 type), and red (HIV-2 type) letters/lines (see **B**). **(B)** Three types of the HIV/SIV genome organization. Genome structures are schematically shown. Letters in boldface type on the right show the lineages analyzed in this study. For virus designations, see Section “Materials and Methods.”

Human immunodeficiency virus types 1 and 2 (HIV-1 and HIV-2) are believed to be generated by extensive cross-species and/or intra-species transmissions of naturally occurring lentiviruses in African primates (Sharp and Hahn, 2011). To date, more than 40 primate species in Africa have been reported to harbor lentiviruses, structurally similar to HIV-1 and HIV-2 (Sharp and Hahn, 2011). Although the evolution and phylogeny of these viruses have been shown to be complicated (Sharp and Hahn, 2011; Shaw and Hunter, 2012; Swanstrom and Coffin, 2012), there are currently eight main lineages in HIV/simian immunodeficiency viruses (SIVs) (Peeters and Courgnaud, 2002; Gordon et al., 2005) (**Figure 1A**). The genomes of various HIV/SIVs individually contain a unique set of accessory genes designated *nef*, *vif*, *vpu*, *vpr* and *vpx* (**Figure 1B**). Accessory proteins encoded by these genes mainly function to inactivate host restriction factors, and thus optimize viral replication (Blanco-Melo et al., 2012; Harris et al., 2012; Malim and Bieniasz, 2012; Simon et al., 2015). While all HIV/SIVs commonly have *nef*, *vif* and *vpr* genes, *vpu* and *vpx* genes are unique to some viral lineages. Upon directing at the *vpx*, *vpr*, and *vpu* genes, various HIV/SIVs can be grouped into three types (Fujita et al., 2010) (**Figure 1B**): prototype viruses with *vpr* only; HIV-1 type viruses carrying *vpr* and *vpu*; HIV-2 type viruses carrying *vpr* and *vpx*. Thus, *vpx* and *vpu* are unique to HIV-2 type and HIV-1 type viruses, respectively. Of note, Vpr and Vpx proteins show significant structural and functional similarities (Khamsri et al., 2006; Ayinde et al., 2010; Fujita et al., 2010). Among the major HIV/SIV lineages, viruses of the two groups, i.e., HIV-2/SIVsmm/stm/mac and SIVrcm/SIVdrl/mnd-2 (**Figure 1A**), have both Vpx and Vpr (**Figure 1B**).

Vpx is a virion-associated protein of 12–16 kDa, and exerts its function in the early stage of infection. Without functional Vpx, HIV-2 type viruses are unable or impeded to grow in natural target cells (Fujita et al., 2010). Recently, a cellular anti-viral factor SAMHD1 has been identified as target for Vpx (Hrecka et al., 2011; Laguette et al., 2011). However, a SAMHD1-independent mechanism(s) is still likely to exist (Fujita et al., 2012; Nomaguchi et al., 2014a; Schaller et al., 2014). From a structural point of view, although Vpx and Vpr are closely related and comprise three helices as described above, they are distinct from each other. Vpx has a zinc finger motif that stabilizes the helical structure (Schwefel et al., 2014), which is not present in Vpr. Notably, there is a well-conserved poly-proline motif (PPM), consisting of seven consecutive prolines, at the C-terminus of HIV-2 and SIVmac Vpx proteins (Miyake et al., 2014a). We previously showed that an HIV-2 mutant virus carrying multi-substitutional mutations in the PPM sequence did not grow at all in human macrophages and grew much more poorly than wild-type (WT) virus in a simian T-cell line, exactly like a Vpx-minus mutant (Fujita et al., 2008). Subsequent molecular studies demonstrated that PPM enhanced Vpx expression at a translation level, not influencing the stability of the protein (Miyake et al., 2014a,b). Our previous work also showed that HIV-1 and HIV-2 Vpr proteins were expressed at a much lower level relative to HIV-2 Vpx, and that the expression level of the two Vpr proteins was not enhanced significantly by simply adding the HIV-2 Vpx PPM sequence (Miyake et al., 2014a). Furthermore, despite a high overall homology of HIV-2 Vpx and SIVmac Vpx, their expression levels in transfected cells were significantly different (Miyake et al., 2014a).

In this report, we performed a linkage study between the Vpx expression profiles and viral phylogeny. Expression plasmids for a wide variety of Vpx proteins derived from diverse primate immunodeficiency viruses (**Figure 1**) were constructed, and monitored for their expression levels and PPM-dependency on the protein expression in transfected cells using SIVagm Vpr proteins as comparative controls. In parallel, phylogenetic trees based on Vpx/Vpr and Gag amino acid sequences were constructed to determine viral evolutionary relationships. The results obtained show that each viral lineage has its characteristic expression property, suggesting a link between the Vpx/Vpr expression pattern and viral evolutionary position.

## Materials and Methods

### Virus Origins

Origins of SIVs are as follows (see also **Figure 1**). Prototype viruses: SIVagm (isolated from the African green monkey); SIVmnd-1 (mandrill); SIVlst (l’Hoest’s monkey); SIVsun (sun-tailed monkey); SIVsyk (Sykes’ monkey); SIVdeb (DeBrazza’s monkey); SIVtal (talapoin monkey); SIVasc (red-tailed guenon); SIVcol (colobus monkey); SIVwrc (western red colobus); SIVolc (olive colobus); SIVkrc (Kibale red colobus). HIV-1 type viruses: SIVcpz (chimpanzee); SIVgor (gorilla); SIVgsn (greater spot-nosed monkey); SIVmon (mona monkey); SIVmus (mustached monkey); SIVden (Dent’s monkey). HIV-2 type viruses: SIVsmm (sooty mangabey monkey); SIVmac (macaque monkey); SIVstm (stump-tailed macaque); SIVrcm (red-capped mangabey); SIVmnd-2 (mandrill); SIVdrl (drill monkey).

### Plasmids

**FIGURE 2:**
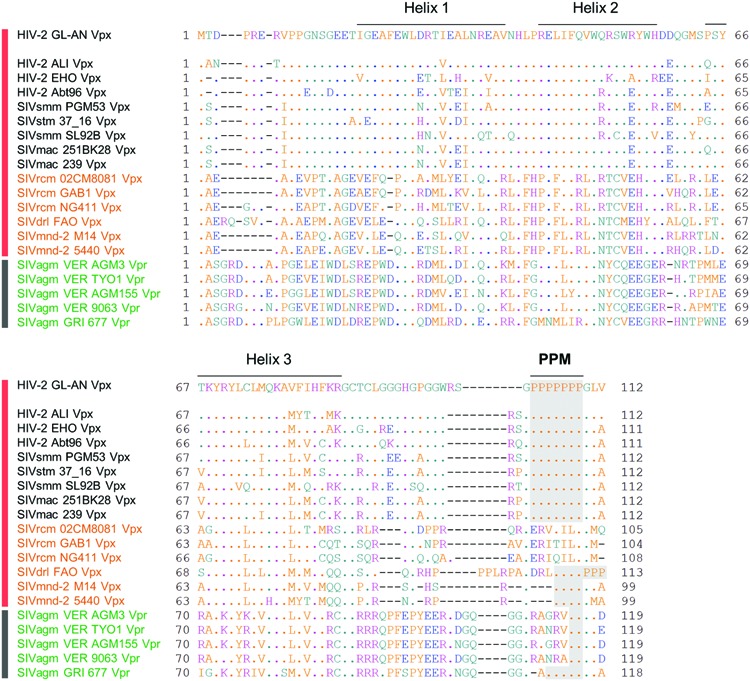
**Sequence alignment of various Vpx/Vpr proteins.** Amino acid sequences of various Vpx/Vpr proteins analyzed in this study are shown. Various Vpx/Vpr proteins on the left derived from the HIV-2/SIVsmm/stm/mac, SIVrcm/SIVdrl/mnd-2, and SIVagm groups (**Figure 1A**) are represented by black, orange, and green letters, respectively. Viruses with HIV-2 type (HIV-2/SIVsmm/stm/mac and SIVrcm/SIVdrl/mnd-2 in **Figure 1B**) and prototype virus (SIVagm in **Figure 1B**) genomes are also indicated by vertical red and black bars, respectively. Sequences were obtained from the HIV sequence database at Los Alamos National Laboratory (http://www.hiv.lanl.gov) and aligned by Genetyx Ver. 11. Locations of three helices of HIV-2 GL-AN Vpx based on the references (Schaller et al., 2014; Schwefel et al., 2014) and a C-terminal PPM (two or more consecutive proline residues) region are indicated as shown.

FLAG-tagged pEF-F expression plasmids for HIV-2 GL-AN Vpx (Genbank accession no., M30895), SIVmac239 Vpx (M33262), and their d7P (a complete deletion of seven consecutive prolines) mutants have been previously described (Miyake et al., 2014a). To generate new FLAG-tagged expression plasmids for Vpx/Vpr proteins in this study, *vpx* and *vpr* genes were synthesized (GenScript) and cloned into pEF-F as described above. New Vpx and Vpr proteins analyzed in this study are as follows (see also **Figure 2**): HIV-2 ALI Vpx (AF082339); HIV-2 EHO Vpx (U27200); HIV-2 Abt96 Vpx (AF208027); SIVsmm PGM53 Vpx (AF077017); SIVstm 37_16 Vpx (M83293); SIVsmm SL92B Vpx (AF334679); SIVmac 251BK28 Vpx (M19499); SIVrcm 02CM8081 Vpx (HM803689); SIVrcm GAB1 Vpx (AF382829); SIVrcm NG411 Vpx (AF349680); SIVdrl FAO Vpx (AY159321); SIVmnd-2 M14 Vpx (AF328295); SIVmnd-2 5440 Vpx (AY159322); SIVagm VER AGM3 Vpr (M30931); SIVagm TYO1 Vpr (DJ048201); SIVagm VER AGM155 Vpr (M29975); SIVagm VER 9063 Vpr (L40990); SIVagm GRI 677 Vpr (M66437). PPM-deletion mutants were constructed by the QuikChange site-directed mutagenesis kit (Agilent Technologies) or by overlap extension PCR using WT clones as templates.

### Transfection

Human kidney 293T cells used for transfection experiments were cultured and maintained as previously described (Miyake et al., 2014a). For transfection, 4.0 μg of each plasmid DNA was introduced into 293T cells by 9.0 μl of Lipofectamine 2000 (Thermo Fisher Scientific).

### Western Blotting

Western blot analysis of transfected cell lysates using anti-FLAG M2 antibody (Sigma) or anti-β-actin AC-15 antibody (Sigma) was conducted as described previously (Miyake et al., 2014a,b). Briefly, supernatants of cell lysates were prepared at 24 h post-transfection, and normalized for total protein amounts by the DC protein assay (Bio-Rad). Samples were then separated on any kD Mini-PROTEAN^®^ TGX^TM^ Precast Gels (Bio-Rad), and transferred onto PVDF membranes (Immobilon-P, Millipore). Immunoreactive viral and cellular proteins were visualized by chemiluminescence using Pierce Western Blotting Substrate Plus (Thermo Fisher Scientific). Experiments were performed at least three times, and representative results are shown. For quantification of the protein band intensities, a GS-800 calibrated densitometer and Quantity One software (Bio-Rad) were used. Mean values ± SD were obtained from at least three independent transfection experiments using HIV-2 GL-AN Vpx as a control.

### Phylogenetic Analysis

Vpx, Vpr, and Gag proteins of HIV/SIVs were phylogenetically analyzed as previously described (Miyake et al., 2014a). Vpr and Gag proteins of SIVsyk were used as references. Amino acid sequences of Vpx, Vpr and Gag proteins deposited in the HIV sequence database at Los Alamos National Laboratory^1^ were aligned by the CLUSTAL_X 2.0.11 program (Thompson et al., 1997; Jeanmougin et al., 1998). Phylogenetic trees were generated by the neighbor-joining method using CLUSTAL_X 2.0.11 program, and branch significance was analyzed by bootstrap with 1000 replicates. Phylogenetic trees were visualized by GENETYX-Tree 2.2.2 program (Genetyx).

## Results

### Sequence Features of Various Vpx Proteins

Through molecular and comparative analyses on Vpx proteins of HIV-2 GL-AN and SIVmac 239 clones, we previously identified two unique regions in Vpx that are important for Vpx expression in cells (Miyake et al., 2014a). One is PPM at the C-terminus and another is helix 1 in the N-terminal region. Deletion of the poly-proline stretch and/or introduction of multi-substitution mutations into the region greatly reduced the expression level of the two Vpx proteins in transfected cells relative to that of parental clones. Based on the observation that HIV-2 GL-AN and SIVmac 239 produce Vpx at a readily distinguishable level upon transfection, we made chimeric clones between the two to locate the determinant sequences that influence the expression level. The responsible region was mapped to four amino acids in the helix 1. We were interested in extending our previous study by monitoring the basal expression level and the PPM-dependency of Vpx proteins from diverse HIV/SIVs. We selected 15 viruses from various HIV/SIVs in the Los Alamos data base to represent the lineages carrying Vpx proteins (HIV-2/SIVsmm/stm/mac and SIVrcm/SIVdrl/mnd-2 groups in **Figure 1**) for analysis in this study (**Figure 2**). Three to five test clones that have no ambiguities in Vpx amino acid sequences were carefully chosen for each subgroup (HIV-2, SIVsmm/stm/mac, SIVrcm, and SIVdrl/mnd-2 in **Figure 1**) to minimize selection biases in the analysis. We focused on examining the Vpx expression here, but five SIVagm Vpr proteins were included because SIVagm Vpr was suggested as the origin of SIVsmm Vpx (Sharp et al., 1996). As readily observed in **Figure 2**, C-terminal PPM is well-conserved among the HIV-2/SIVsmm/stm/mac group and a SIVdrl/mnd-2 subgroup in the SIVrcm/SIVdrl/mnd-2 group (**Figure 1A**). However, no PPM is present at the corresponding region of Vpx proteins from another subgroup (SIVrcm) in the SIVrcm/SIVdrl/mnd-2 group (**Figure 1A**). Notably, there is a clear PPM consisting of five consecutive prolines in Vpr of an SIVagm strain (GRI 677) (**Figure 2**). Another point worth mentioning here is that Vpx/Vpr proteins are quite miscellaneous among the lineages, and there are scattered amino acid differences even in the helix region of the proteins from the same viral lineage (**Figure 2**). This is true for the helix 1 region of Vpx proteins in the HIV-2/SIVsmm/stm/mac group (**Figure 2**).

### Expression Profiles for Various Vpx/Vpr Proteins

**FIGURE 3:**
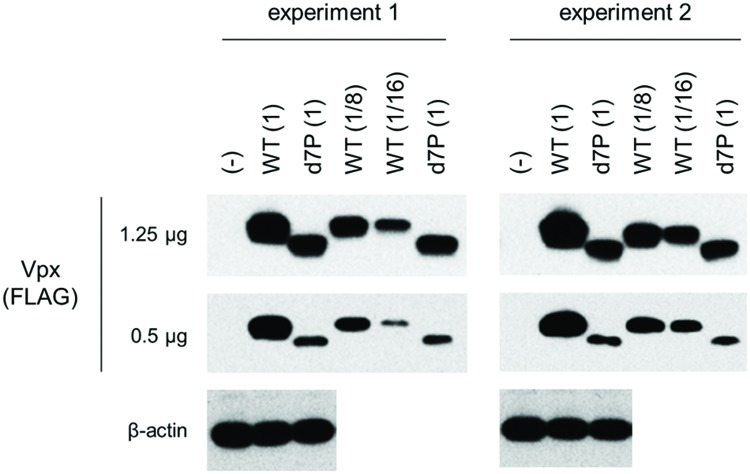
**Expression levels of HIV-2 GL-AN Vpx and its d7P mutant.** Expression levels of wild-type (WT) and mutant Vpx proteins in transfected 293T cells were examined by Western blotting analysis. Results of two independent experiments are shown. Relative protein amounts used for blotting are indicated in parentheses. As WT samples, an eighth and a sixteenth amounts were also used for comparative quantification purpose. (-), pEF1/*Myc*-HisA (empty vector); WT, pEF-Fvpx; d7P, pEF-Fvpx/d7P mutant (Miyake et al., 2014a,b).

We assumed, based on empirical knowledge (Khamsri et al., 2006; Fujita et al., 2008; Miyake et al., 2014a), that some Vpx/Vpr proteins derived from diverse HIV/SIVs would be produced at a very low level upon transfection. In order to perform a systemic quantitative analysis on the expression of these proteins, especially to detect and compare a minimal level expression, a highly efficient transfection method generating highly reproducible results is required. Although we employed the calcium-phosphate co-precipitation method in previous studies (Miyake et al., 2014a,b), we selected here to use the lipofection method instead because of its better reproducibility. First, we re-evaluated, by this new method, the effect of PPM-deletion on the Vpx expression level of HIV-2 in a quantitative manner. Parental and mutant clones were transfected into 293T cells, and 24 h later, sample cell lysates were prepared for Western blot analysis. As is clear in **Figure 3**, the d7P mutant expressed mutant Vpx protein at a level between an eighth and a sixteenth relative to that by WT clone. The d7P mutant protein was readily detected in both experiments, and the PPM-dependent expression of HIV-2 Vpx was evident in both experiments.

**FIGURE 4:**
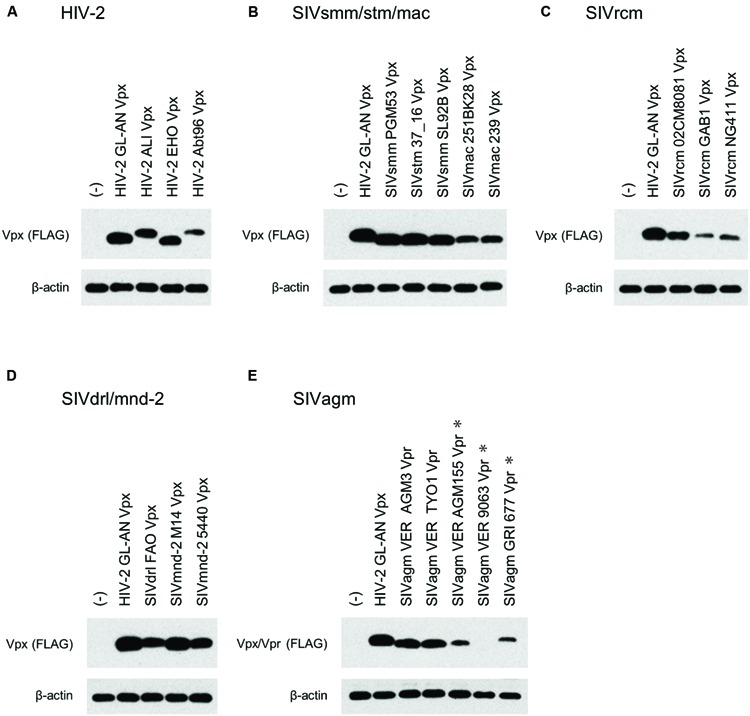
**Expression of Vpx/Vpr proteins from various HIV/SIVs.** Expression of various Vpx/Vpr proteins **(A–E)** in transfected 293T cells as monitored by Western blotting analysis is shown. Viral groups (**Figure 1A**) are indicated. ^∗^, 10-fold more protein amounts were used to detect Vpr, but same protein amounts with those for the other four lanes were used to detect β-actin here. (-), pEF1/*Myc*-HisA (empty vector).

**FIGURE 5:**
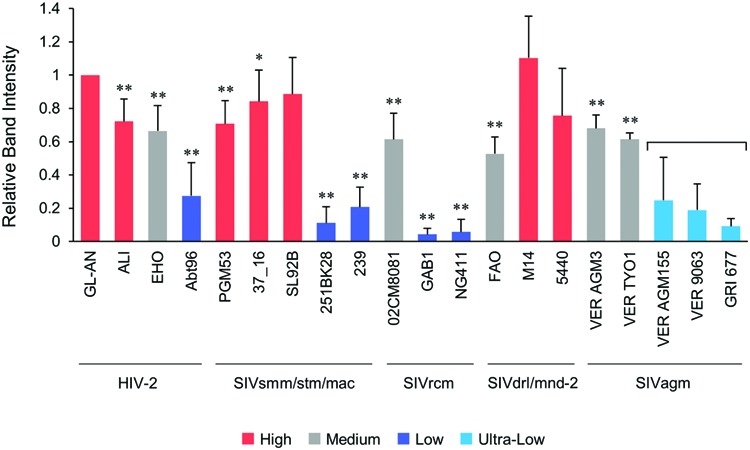
**Quantification of the Vpx/Vpr expression levels.** Following visualization of blotted Vpx/Vpr proteins, the band intensities were determined by a densitometer, and the expression levels relative to that of HIV-2 GL-AN Vpx were calculated. Based on the mean intensity values, the expression levels were grouped into High (>70% relative to GL-AN), Medium (30–70%), Low (<30%), and Ultra-Low (undetectable under the standard condition). For Ultra-Low group, 10-fold more protein amounts were used to perform Western blotting analysis. Viral groups (**Figure 1A**) are indicated under the lines. Mean values ± SD from at least three independent experiments are shown. Significance relative to HIV-2 GL-AN as calculated by the Student *t*-test is shown (^∗^*P* < 0.05; ^∗∗^*P* < 0.01).

We then comparatively and quantitatively assessed the expression levels of numerous Vpx and Vpr proteins derived from a variety of HIV/SIVs. Eighteen new expression plasmids with a FLAG-tag were constructed (**Figure 2**), and examined for their expression in 293T cells following transfection. HIV-2 (GL-AN) Vpx was used as a control throughout the experiments. **Figure 4** shows the representative results obtained by this all-inclusive monitoring. As predicted, the Vpx/Vpr expression levels, by viral clones belonging to the different groups (**Figures 4A–E** correspond to the HIV-2, SIVsmm/stm/mac, SIVrcm, SIVdrl/mnd-2, and SIVagm groups, respectively), significantly or clearly varied. In addition, Vpx and Vpr proteins were produced at a different level even by viruses within the same group, and no clear group-specificity with respect to the Vpx/Vpr expression level in cells was observed. Of note, small differences were observed for Vpx proteins from the SIVdrl/mnd-2 group (**Figure 4D**). Remarkably, some SIVagm Vpr proteins were expressed at an extremely low level (**Figure 4E**). To better substantiate the results in **Figure 4**, we quantified the expression levels by densitometric monitoring of the Vpx/Vpr band intensities, and calculated the levels relative to that by the control HIV-2 GL-AN. As shown in **Figure 5**, the expression levels could be categorized into four groups: high (>70% relative to GL-AN), medium (30–70%), low (<30%), and ultra-low (minimum expression). Overall, these results quantitatively confirmed that the expression levels of Vpx/Vpr vary considerably among primate lentiviruses.

**FIGURE 6:**
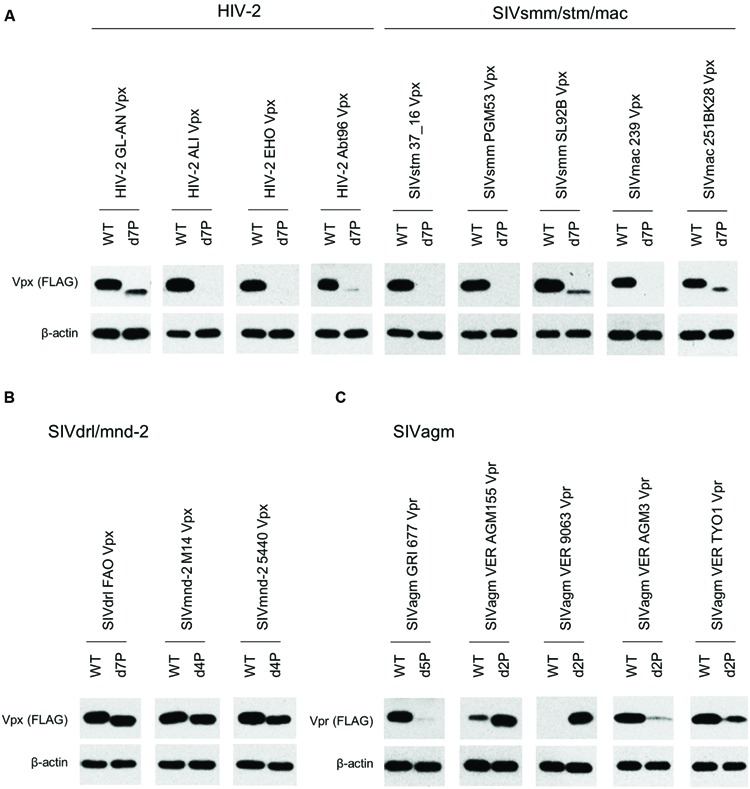
**Expression of PPM-deleted Vpx/Vpr mutant proteins from various HIV/SIVs.** Expression of PPM-deleted Vpx/Vpr proteins **(A–C)** in transfected 293T cells as monitored by Western blotting analysis is shown. Viral groups (**Figure 1A**) are indicated. Each deletion mutant (d2P, d4P, d5P, and d7P) lacks the entire PPM sequence (**Figure 2**). Amounts of sample cell lysates, antibody concentrations, and exposure time were adjusted individually for appropriate band intensity as shown.

In order to determine the PPM dependency of Vpx/Vpr expression by clones containing PPM (two or more consecutive prolines), we constructed PPM-deletion mutants from various virus species (**Figure 2**), and examined their expressions relative to parental clones. **Figure 6** shows the results obtained for each viral group: HIV-2/SIVsmm/stm/mac in panel A; SIVdrl/mnd-2 in B; SIVagm in C. As clearly observed, most Vpx/Vpr proteins exhibited the PPM-dependency except for those from viruses in the SIVdrl/mnd-2 group (**Figure 6B**). Unexpectedly, the expression levels of Vpr proteins derived from the two virus strains in the SIVagm group were enhanced by PPM-deletion (**Figure 6C**), in a sharp contrast to the results for the others. In total, the results in **Figure 6** revealed that the PPM-dependent expression of Vpx, including SIVagm Vpr, is a conserved feature among most HIV-2/SIVs in the transfected 293T cells.

### Phylogenetic Study

**FIGURE 7:**
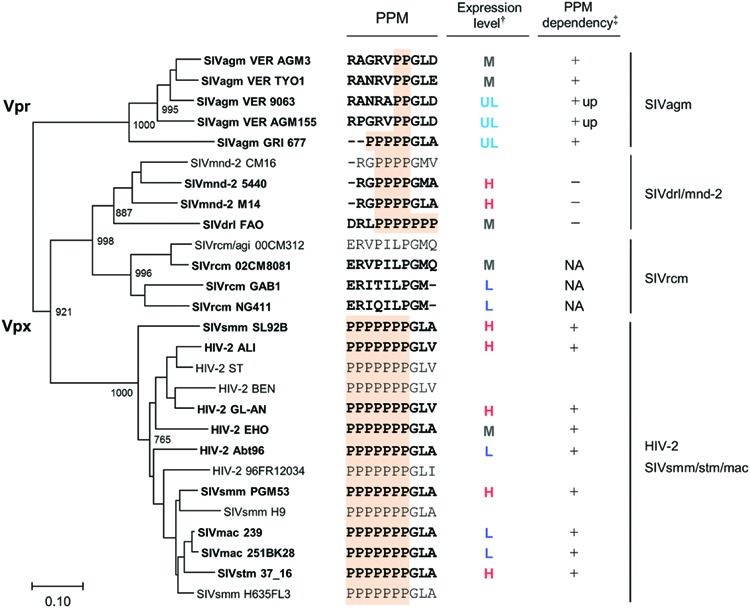
**Phylogeny and expression profiles of Vpx/Vpr proteins.** Phylogenetic tree based on Vpx/Vpr proteins and their expression profiles in cells are shown in parallel (see **Figure 1A** for viral groups on the right). Scale bar represents the genetic distance. Branches were calculated from 1000 bootstrap replicates, and the bootstrap values are labeled on the major branches. Viral strains experimentally analyzed in this study (20 strains) are highlighted by boldface type letters. C-terminal regions of Vpx/Vpr proteins including PPM sequence (highlighted) and the summarized expression profiles (**Figures 4–6**) are shown in the middle. †: H, high; M, medium; L, low; UL, ultra-low (see **Figure 5**). ‡: +, decreased expression by PPM-deletion; +up, increased expression by PPM-deletion; -, no clear effect by PPM-deletion; NA, not applicable (no PPM).

We constructed a phylogenetic tree of various Vpx/Vpr proteins to determine whether the expression profiles of viral Vpx/Vpr proteins presented so far could be related to viral evolutional positions (**Figure 7**). As is recognizable in this figure, evolutional group-dependent properties for the Vpx/Vpr expression pattern became clear. While members in the SIVdrl/mnd-2 group expressed a relatively high level of Vpx without PPM-dependence, clones in the SIVrcm group do not have the PPM itself. Although the Vpx expression levels observed for the SIVrcm clones were significantly different, this could be a subgroup difference. In the large HIV-2/SIVsmm/stm/mac group, all clones share the C-terminal seven consecutive prolines, and all the clones examined exhibited the PPM-dependency for their Vpx expression. Considering the branching to subgroups, the distinct expression levels from (L) to (H) in this large group may be evolutionarily explainable. As observed in the phylogenetic tree (**Figure 7**), some viruses in one subgroup may have acquired or lost their properties in the course of adaptation and evolution to form the other subgroups (for example, see SIVsmm SL92B, HIV-2 EHO, and HIV-2 Abt96). Finally, two strains (VER 9063 and VER AGM155) in the SIVagm group that show unique PPM-dependency were positioned relatively close to each other within the group. In total, our results here suggest a possible link between the expression profiles (the basal expression level and PPM-dependency in the transfected 293T cells) of Vpx/Vpr proteins and the primate lentiviral evolutional positions.

**FIGURE 8:**
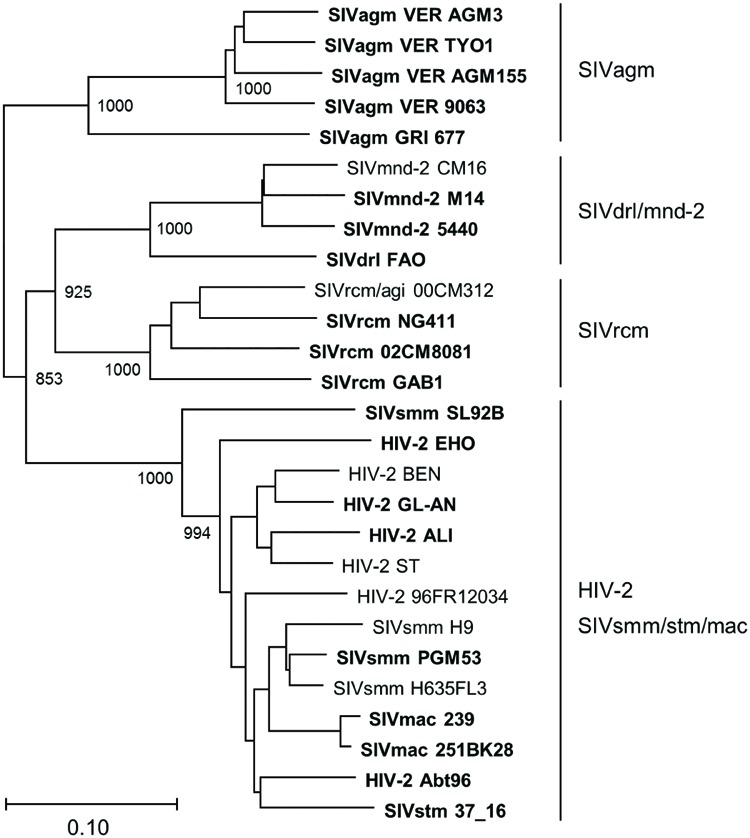
**Phylogeny of Gag proteins.** Phylogenetic tree based on Gag proteins is shown. Viral groups (**Figure 1A**) are indicated on the right. Viral strains experimentally analyzed in this study (20 strains) are highlighted by boldface type letters. Scale bar indicates the genetic distance. Branches were calculated from 1000 bootstrap replicates, and the bootstrap values are labeled on the major branches.

The phylogenetic tree described above was based on Vpx/Vpr amino acid sequences. Therefore, it was possible that our experimental data on various HIV/SIVs (**Figures 4–6**) simply reflected the amino acid sequence similarity *per se*, not being indicative of accurate evolutionary history. To exclude this possibility, we constructed a phylogenetic tree based on major structure protein Gag, and compared the two phylogenetic trees. As is clear in **Figure 8**, the branching pattern in Gag-tree was generally consistent with that in Vpx/Vpr-tree (**Figure 7**), ruling out the above possibility.

## Discussion

In this study, we constructed numerous expression plasmids for diverse Vpx/Vpr proteins derived from HIV-2 type and prototype viruses (**Figure 1**), and examined their basal and PPM-dependent expression phenotypes in cells (**Figure 4**–**6**). We also constructed phylogenetic trees based on viral Vpx/Vpr and Gag proteins (**Figures 7** and **Figure 8**). Basal expression levels of Vpx/Vpr proteins were found to be highly variable, but appeared to be evolutional group-dependent (**Figures 7** and **Figure 8**). PPM-dependent or -independent expression of Vpx/Vpr was demonstrated to be evolutional group-specific (**Figure 7** and **8**). Taken together, it is not unreasonable to conclude that various HIV/SIVs may have acquired characteristic abilities to express Vpx/Vpr proteins to meet with their surrounding circumstances. This is the first report that suggests the evolutional significance of Vpx/Vpr expression patterns.

Two strains in the SIVsmm/stm/mac group analyzed here (SIVmac 239 and 251BK28) expressed Vpx at a low level, whereas closely related viruses of this group all produced Vpx at a high level (SIVsmm PGM53 and SL92B, and SIVstm 37_16) (**Figure 5**). Probably, the low level phenotype was newly acquired by SIVmac when branched from SIVsmm. Similarly, viral strains GAB1 and NG411 in the SIVrcm group expressed Vpx at a low level, but another member 02CM8081 did at a medium level (**Figure 5**). Because GAB1 and NG411 cluster together to form a separate subgroup from the 02CM8081 group on the phylogenetic tree (**Figure 7**), one can postulate that different expression phenotypes were independently acquired. Viruses in the SIVrcm group are particularly interesting, for only they carry Vpx without PPM. Highly divergent sequences of SIVrcm Vpx (Beer et al., 2001) might be associated with this unique feature. Whereas SIVsmm Vpx has PPM of seven-consecutive prolines not found in SIVrcm Vpx, SIVrcm and SIVsmm were suggested to gain their Vpx proteins before their divergence (Etienne et al., 2013). Therefore, it is intriguing to elucidate why and how these two viruses came to encode distinct Vpx proteins with/without PPM.

The SIVdrl/SIVmnd-2 group exceptionally lacks PPM-dependency for its Vpx expression (**Figure 6**). Viruses in this group have a relatively long PPM (four or seven consecutive prolines) (**Figure 2**) and expressed Vpx at a medium or high level (**Figure 5**). SIVdrl and SIVmnd-2 have mosaic genome structures and were suggested to have arisen from a recombinational event(s) between SIVrcm with *vpx* and SIVmnd-1 without *vpx* (**Figure 1**) (Takemura and Hayami, 2004). Therefore, Vpx proteins of SIVdrl and SIVmnd-2 are predicted to be originated from an ancient SIVrcm Vpx. Because SIVrcm Vpx lacks PPM (**Figure 2**), one needs to assume that SIVdrl and SIVmnd-2 newly acquired Vpx with PPM in the evolution process. Or, it could be supposed that there was PPM in the ancient SIVrcm Vpx, and that the PPM was subsequently lost from the Vpx (after divergence of SIVdrl and SIVmnd-2 from SIVrcm).

Remarkably, two SIVagm Vpr proteins (VER 9063 and VER AGM155) were expressed at a faint level in cells, and have a di-proline motif that suppresses protein expression (**Figure 7**). While two other SIVagm Vpr proteins (VER AGM3 and VER TYO1) exhibited an ordinary phenotype (medium level expression and PPM-dependency), the fifth one (GRI 677) showed a unique character, i.e., ultra-low level expression and PPM-dependency, in the group (**Figure 7**). Viruses in the SIVagm group were reported to have a greater genetic diversity compared to other primate lentiviruses (Johnson et al., 1990), in agreement with a complex phenotype observed in this study as described above (**Figure 7**). SIVagm *vpr* gene was initially categorized as *vpx* due to its apparent sequence similarity to HIV-2 and SIVsmm *vpx* genes. However, it was subsequently reclassified as *vpr*, and also was proposed as the origin of SIVsmm *vpx* (Sharp et al., 1996). The *vpx* gene is found only in HIV-2 type viruses (**Figure 1**) and all these viruses were presumably originated from SIVagm. Thus, it can be assumed that SIVagm Vpr with its own PPM sequence evolved into various Vpx proteins. Recently, a similar hypothesis was presented for the SAMHD1 counteraction by Vpx and Vpr proteins (Lim et al., 2012). In the report, phylogeny-based observation has suggested that the SAMHD1 antagonism by *vpr* preceded the appearance of *vpx*.

A high divergence in the expression profiles observed for Vpx/Vpr proteins is virologically important. Various HIV/SIVs appeared to express Vpx/Vpr proteins at levels of an unexpectedly wide range (more than 100-fold difference) (**Figures 4, 5** and **7**). We previously reported that the helix 1 (four amino acids) was a determinant for the Vpx expression level (Miyake et al., 2014a). However, taking account of considerably variable amino acid sequences in the helix 1 (**Figure 2**) of Vpx proteins with low and high expression phenotypes (**Figures 4, 5** and **7**), we cannot simply conclude now that the helix 1 is responsible for determining the expression levels. In addition, some Vpx/Vpr proteins are PPM-dependent and others are PPM-independent for their expressions (**Figures 6** and **7**). Furthermore, there is no PPM in some Vpx proteins derived from one viral group. Considering that Vpx/Vpr proteins are very likely to be important for HIV/SIV replication, persistence and/or transmission in natural primate hosts, the heterogeneity with respect to the expression level of a viral protein is remarkable. However, of note, the phylogeny of various HIV/SIVs generally shows a cluster pattern similar with that of the natural hosts (Perelman et al., 2011), supporting the notion that HIV/SIVs have acquired various expression profiles of Vpx/Vpr in the course of virus diversification. Although presently unknown, biological and molecular bases/reasons for the observations described above must exist. Our work reported here would evoke studies to determine the processes and underlying molecular bases (transcription, translation, stability of Vpx/Vpr, cytotoxicity of Vpx/Vpr, responsible sequence or structure of Vpx/Vpr, relevant cellular factors, and so on) for the observed heterogeneity among various HIV/SIVs. In this regard, of note, our recent studies have demonstrated that HIV-1 can adapt itself to various APOBEC3G environments by regulating the Vif expression level (Nomaguchi et al., 2014b, 2016).

Although we demonstrate here that Vpx/Vpr expression profiles are potentially linked to the phylogeny of various HIV/SIVs, our research system used was rather artificial, and how the Vpx/Vpr proteins contribute to the biology of HIV/SIVs in host individuals and populations remains to be elucidated. Further studies utilizing infectious intact proviral clones derived from various HIV/SIVs and natural target cells from various primate hosts are required to reveal the biological role for Vpx/Vpr in the process of viral adaptation and evolution.

## Author Contributions

YS: acquisition, analysis, and interpretation of data for the work; drafting the work; final approval of the manuscript. AM: acquisition, analysis, and interpretation of data for the work; final approval of the manuscript. ND: acquisition, analysis, and interpretation of data for the work; final approval of the manuscript. HS: acquisition, analysis, and interpretation of data for the work; final approval of the manuscript. YM: acquisition, analysis, and interpretation of data for the work; final approval of the manuscript. AA: design of the work; analysis, and interpretation of data for the work; drafting the work; final approval of the manuscript agreement to be accountable for all aspects of the work. MN: design of the work; analysis, and interpretation of data for the work; drafting the work; final approval of the manuscript agreement to be accountable for all aspects of the work.

## Conflict of Interest Statement

The authors declare that the research was conducted in the absence of any commercial or financial relationships that could be construed as a potential conflict of interest.

## Footnotes

1 http://www.hiv.lanl.gov

## References

[B1] Ayinde D. Maudet C. Transy C. Margottin-Goguet F. (2010). Limelight on two HIV/SIV accessory proteins in macrophage infection: is Vpx overshadowing Vpr? *Retrovirology* 7:35 10.1186/1742-4690-7-35 PMC2867959 20380700

[B2] Beer B. E. Foley B. T. Kuiken C. L. Tooze Z. Goeken R. M. Brown C. R. (2001). Characterization of novel simian immunodeficiency viruses from red-capped mangabeys from Nigeria (SIVrcmNG409 and -NG411). *J. Virol.* 75 12014–12027. 10.1128/JVI.75.24.12014-12027.2001 11711592 PMC116097

[B3] Blanco-Melo D. Venkatesh S. Bieniasz P. D. (2012). Intrinsic cellular defenses against human immunodeficiency viruses. *Immunity* 37 399–411. 10.1016/j.immuni.2012.08.013 22999946 PMC3912573

[B4] Etienne L. Hahn B. H. Sharp P. M. Matsen F. A. Emerman M. (2013). Gene loss and adaptation to hominids underlie the ancient origin of HIV-1. *Cell Host Microbe* 14 85–92. 10.1016/j.chom.2013.06.002 23870316 PMC3733229

[B5] Fujita M. Nomaguchi M. Adachi A. Otsuka M. (2012). SAMHD1-dependent and -independent functions of HIV-2/SIV Vpx protein. *Front. Microbiol.* 3:297 10.3389/fmicb.2012.00297 PMC3415948 22908011

[B6] Fujita M. Otsuka M. Nomaguchi M. Adachi A. (2008). Functional region mapping of HIV-2 Vpx protein. *Microbes Infect.* 10 1387–1392. 10.1016/j.micinf.2008.08.005 18771746

[B7] Fujita M. Otsuka M. Nomaguchi M. Adachi A. (2010). Multifaceted activity of HIV Vpr/Vpx proteins: the current view of their virological functions. *Rev. Med. Virol.* 20 68–76. 10.1002/rmv.636 20069611

[B8] Gordon S. Pandrea I. Dunham R. Apetrei C. Silvestri G. (2005). “The call of the wild: what can be learned from studies of SIV infection of natural hosts?” in *HIV Sequence Compendium*, eds Thomas Leitner T. Foley B. Hahn B. Marx P. McCutchan F. Mellors J. (Los Alamos, NM: Theoretical Biology and Biophysics Group, Los Alamos National Laboratory).

[B9] Harris R. S. Hultquist J. F. Evans D. T. (2012). The restriction factors of human immunodeficiency virus. *J. Biol. Chem.* 287 40875–40883. 10.1074/jbc.R112.416925 23043100 PMC3510791

[B10] Hrecka K. Hao C. Gierszewska M. Swanson S. K. Kesik-Brodacka M. Srivastava S. (2011). Vpx relieves inhibition of HIV-1 infection of macrophages mediated by the SAMHD1 protein. *Nature* 474 658–661. 10.1038/nature10195 21720370 PMC3179858

[B11] Jeanmougin F. Thompson J. D. Gouy M. Higgins D. G. Gibson T. J. (1998). Multiple sequence alignment with CLUSTAL_X. *Trends Biochem. Sci.* 23 403–405. 10.1016/S0968-0004(98)01285-7 9810230

[B12] Johnson P. R. Fomsgaard A. Allan J. Gravell M. London W. T. Olmsted R. A. (1990). Simian immunodeficiency viruses from African green monkeys display unusual genetic diversity. *J. Virol.* 64 1086–1092. 2304139 10.1128/jvi.64.3.1086-1092.1990 PMC249221

[B13] Khamsri B. Murao F. Yoshida A. Sakurai A. Uchiyama T. Shirai H. (2006). Comparative study on the structure and cytopathogenic activity of HIV Vpr/Vpx proteins. *Microbes Infect.* 8 10–15. 10.1016/j.micinf.2005.05.020 16153874

[B14] Laguette N. Sobhian B. Casartelli N. Ringeard M. Chable-Bessia C. Ségéral E. (2011). SAMHD1 is the dendritic- and myeloid-cell-specific HIV-1 restriction factor counteracted by Vpx. *Nature* 474 654–657. 10.1038/nature10117 21613998 PMC3595993

[B15] Lim E. S. Fregoso O. I. McCoy C. O. Matsen F. A. Malik H. S. Emerman M. (2012). The ability of primate lentiviruses to degrade the monocyte restriction factor SAMHD1 preceded the birth of the viral accessory protein Vpx. *Cell Host Microbe* 11 194–204. 10.1016/j.chom.2012.01.004 22284954 PMC3288607

[B16] Malim M. H. Bieniasz P. D. (2012). HIV restriction factors and mechanisms of evasion. *Cold Spring Harb. Perspect. Med.* 2:a006940 10.1101/cshperspect.a006940 PMC3331687 22553496

[B17] Miyake A. Fujita M. Fujino H. Koga R. Kawamura S. Otsuka M. (2014a). Poly-proline motif in HIV-2 Vpx is critical for its efficient translation. *J. Gen. Virol.* 95 179–189. 10.1099/vir.0.057364-0 24114794

[B18] Miyake A. Miyazaki Y. Fujita M. Nomaguchi M. Adachi A. (2014b). Role of poly-proline motif in HIV-2 Vpx expression. *Front. Microbiol.* 5:24 10.3389/fmicb.2014.00024 PMC3904113 24478770

[B19] Nomaguchi M. Doi N. Adachi A. (2014a). Virological characterization of HIV-2 *vpx* gene mutants in various cell systems. *Microbes Infect.* 16 695–701. 10.1016/j.micinf.2014.06.004 24956595

[B20] Nomaguchi M. Doi N. Sakai Y. Ode H. Iwatani Y. Ueno T. (2016). Natural single-nucleotide variations in the HIV-1 genomic SA1prox region can alter viral replication ability by regulating Vif expression levels. *J. Virol.* 90 4563–4578. 10.1128/JVI.02939-15 26912631 PMC4836328

[B21] Nomaguchi M. Miyake A. Doi N. Fujiwara S. Miyazaki M. Tsunetsugu-Yokota Y. (2014b). Natural single-nucleotide polymorphisms in the 3’ region of the HIV-1 pol gene modulate viral replication ability. *J. Virol.* 88 4145–4160. 10.1128/JVI.01859-13 24478432 PMC3993728

[B22] Peeters M. Courgnaud V. (2002). “Overview of primate lentiviruses and their evolution in non-human primates in Africa,” in *HIV Sequence Compendium*, eds Kuiken C. Foley B. Freed E. Hahn B. Korber B. Marx P. A. (Los Alamos, NM: Theoretical Biology and Biophysics Group, Los Alamos National Laboratory).

[B23] Perelman P. Johnson W. E. Roos C. Seuánez H. N. Horvath J. E. Moreira M. A. M. (2011). A molecular phylogeny of living primates. *PLoS Genet.* 7:e1001342 10.1371/journal.pgen.1001342 PMC3060065 21436896

[B24] Schaller T. Bauby H. Hué S. Malim M. H. Goujon C. (2014). New insights into an X-traordinary viral protein. *Front. Microbiol.* 5:126 10.3389/fmicb.2014.00126 PMC3986551 24782834

[B25] Schwefel D. Groom H. C. Boucherit V. C. Christodoulou E. Walker P. A. Stoye J. P. (2014). Structural basis of lentiviral subversion of a cellular protein degradation pathway. *Nature* 505 234–238. 10.1038/nature12815 24336198 PMC3886899

[B26] Sharp P. M. Bailes E. Stevenson M. Emerman M. Hahn B. H. (1996). Gene acquisition in HIV and SIV. *Nature* 383 586–587. 10.1038/383586a0 8857532 PMC9514223

[B27] Sharp P. M. Hahn B. H. (2011). Origins of HIV and the AIDS pandemic. *Cold Spring Harb. Perspect. Med.* 1:a006841 10.1101/cshperspect.a006841 PMC3234451 22229120

[B28] Shaw G. M. Hunter E. (2012). HIV transmission. *Cold Spring Harb. Perspect. Med.* 2:a006965 10.1101/cshperspect.a006965 PMC3543106 23043157

[B29] Simon V. Bloch N. Landau N. R. (2015). Intrinsic host restrictions to HIV-1 and mechanisms of viral escape. *Nat. Immunol.* 16 546–553. 10.1038/ni.3156 25988886 PMC6908429

[B30] Swanstrom R. Coffin J. (2012). HIV-1 pathogenesis: the virus. *Cold Spring Harb. Perspect. Med.* 2:a007443 10.1101/cshperspect.a007443 PMC3543077 23143844

[B31] Takemura T. Hayami M. (2004). Phylogenetic analysis of SIV derived from mandrill and drill. *Front. Biosci.* 9:513–520. 10.2741/1242 14766387

[B32] Thompson J. D. Gibson T. J. Plewniak F. Jeanmougin F. Higgins D. G. (1997). The CLUSTAL_X windows interface: flexible strategies for multiple sequence alignment aided by quality analysis tools. *Nucleic Acids Res.* 25 4876–4882. 10.1093/nar/25.24.4876 9396791 PMC147148

